# ProSeal laryngeal mask airway: An alternative to endotracheal intubation in paediatric patients for short duration surgical procedures

**DOI:** 10.4103/0019-5049.72644

**Published:** 2010

**Authors:** Jaya Lalwani, Kamta Prasad Dubey, Bal Swaroop Sahu, Pratibha Jain Shah

**Affiliations:** Pt. J.N.M. Medical College & Dr. BRAM Hospital, Raipur (C.G.), India

**Keywords:** ET tube, haemodynamic response, PLMA

## Abstract

The laryngeal mask airway (LMA) is a supraglottic airway management device. The LMA is preferred for airway management in paediatric patients for short duration surgical procedures. The recently introduced ProSeal (PLMA), a modification of Classic LMA, has a gastric drainage tube placed lateral to main airway tube which allows the regurgitated gastric contents to bypass the glottis and prevents the pulmonary aspiration. This study was done to compare the efficacy of ProSeal LMA with an endotracheal tube in paediatric patients with respect to number of attempts for placement of devices, haemodynamic responses and perioperative respiratory complications. Sixty children, ASA I and II, weighing 10-20 kg between 2 and 8 years of age group of either sex undergoing elective ophthalmological and lower abdominal surgeries of 30-60 min duration, randomly divided into two groups of 30 patients each were studied. The number of attempts for endotracheal intubation was less than the placement of PLMA. Haemodynamic responses were significantly higher (*P*<0.05) after endotracheal intubation as compared to the placement of PLMA. There were no significant differences in mean SpO_2_ (%) and EtCO_2_ levels recorded at different time intervals between the two groups. The incidence of post-operative respiratory complications cough and bronchospasm was higher after extubation than after removal of PLMA. The incidence of soft tissue trauma was noted to be higher for PLMA after its removal. There were no incidences of aspiration and hoarseness/sore throat in either group. It is concluded that ProSeal LMA can be safely considered as a suitable and effective alternative to endotracheal intubation in paediatric patients for short duration surgical procedures.

## INTRODUCTION

Children have been the earliest patrons of anaesthesiology from its earliest clinical applications of surgical anaesthesia.[1] An endotracheal tube (ET) is always considered to be the gold standard[2,3] device to maintain an airway because of its inherent ability to provide positive pressure ventilation (PPV) and to prevent occurrences of gastric inflation and pulmonary aspiration.[3] Haemodynamic responses, situations of failed intubation and damage to the oropharyngeal structures[3] during intubation are also a serious concern. The first supraglottic airway device–the Laryngeal Mask Airway (LMA)–was designed in 1981 by Dr. Archie Brain.[4] The paediatric ClassicLMA forms a less effective glottic seal[5] with the subsequent risk of gastric distension and regurgitation due to leakage of gas in the stomach which can lead to pulmonary aspiration.

The ProSeal LMA was introduced by Dr. Archie Brain in 2000.[4] ProSeal LMA has a gastric drainage tube, placed lateral to the main airway tube. The gastric drainage tube forms a channel for regurgitated gastric contents[5] and prevents gastric insufflation and pulmonary aspiration. A gastric tube can be placed through a drain tube and can detect the malposition[5] of PLMA. The paediatric PLMA lacks the dorsal cuff.[5] The paediatric ProSeal LMA available sizes are 1, 1.5, 2 and 2.5.

In the present study we compared the PLMA (size 2) and ET tube with respect to number of attempts for the placement of devices, haemodynamic responses during placement and perioperative respiratory complications.

## METHODS

After approval from institutional ethical committee, written informed consent was taken from all the parents. Sixty children of 2-8 years of age group of either sex, weighing 10-20 kg and belonging to physical status of ASA Grades I and II scheduled for elective ophthalmological and lower abdominal surgical procedures of 30-60 min duration were undertaken for the study. All the patients were randomly divided into two groups by a draw method (simple randomization) of either PLMA (group A) or ET (group B). Patients with the lack of written informed consent anticipated difficult airway, hiatus hernia, gastro-esophageal reflux diseases, cardio respiratory diseases, upper respiratory tract infection (URI), history of convulsions and full stomach were excluded from the study. A thorough preoperative assessment was done a day before surgery.

All patients were premedicated with i.v. Glycopyrrolate 0.004 mg/kg, i.v. Fentanyl 2 *µ*g/kg and i.v. Ondansetron 0.1 mg/kg 5 min prior to induction of anaesthesia. Standard monitoring were applied which included precordial stethoscope, pulse oximeter, capnography, electrocardiography and automated noninvasive blood pressure (NIBP). Base line vital parameters were recorded. After preoxygenation, anaesthesia was induced with i.v. propofol 2 mg/kg mixed with i.v. lignocaine 0.5 mg/kg. Atracurium 0.5 mg/kg was used as neuromuscular blocking agent (NMBA) and intermittent boluses of i.v. atracurium 0.1 mg/kg were given as required.

PLMA size 2 was selected for group A patients, the cuff was fully deflated and posterior surface of PLMA was well lubricated with 2% lignocaine jelly. The child’s head was maintained in the sniffing position. The PLMA was inserted through oral cavity using the index finger technique. The cuff was inflated with 7-10 ml of air as recommended by manufacturer. A number of insertion attempts were recorded. Three attempts were allowed for the placement before the device was considered a failure and the device was replaced with an ET tube. Removal of the device from mouth was termed as failed attempt. After obtaining an effective airway (defined as normal thoracoabdominal movements, bilaterally equal audible breath sounds on auscultation and a regular waveform on capnograph), the PLMA was fixed by taping over the chin. The PLMA position was confirmed by the gel displacement test, bilateral chest movements and square wave capnography. Gastric tube number 10 was inserted through a drain tube. Two attempts were allowed before gastric tube insertion was considered a failure and repositioning of PLMA was done. In group B patients, endotracheal intubation was done using appropriate size cuffed or uncuffed tubes. Uncuffed eETs were preferred for children younger than 6 years.

All patients were maintained on nitrous oxide 66% in oxygen and halothane 0.8-1% and manually ventilated using Jackson Ree’s modification of Ayre’s T-piece. EtCO_2_ was maintained between 35 and 45 mmHg. Immediately after placement of PLMA and ET intubation, vital parameters were recorded. Haemodynamic parameters were recorded at 5 min and 10 min interval after placement of PLMA and intubation. At the end of surgery, anaesthetic agents were discontinued and patients were kept on 100% oxygen and i.v. glycopyrrolate 0.004 mg/kg followed by i.v. neostigmine 0.05 mg/kg for adequate reversal of residual neuromuscular blockade was given. After full deflation of cuff the PLMA was removed in a spontaneously breathing patient. Similarly, extubation was done after thorough oral suction.

During emergence, the occurrence of any complications like coughing, bronchospasm and laryngospasm was noted. After removal of both airway devices, blood staining of the ET tube and posterior aspect of cuff of PLMA, tongue-lip-dental trauma and hoarseness was recorded.

The patients were monitored throughout the perioperative period till stay in the post-anaesthesia care unit. The patients were followed for next 24 h for any sore throat and hoarseness.

For statistical analysis the data were analyzed using the SPSS software (6.0 version). Student’s t test was applied. *P* value <0.05 was considered significant.

## RESULTS

**Figure 1:**
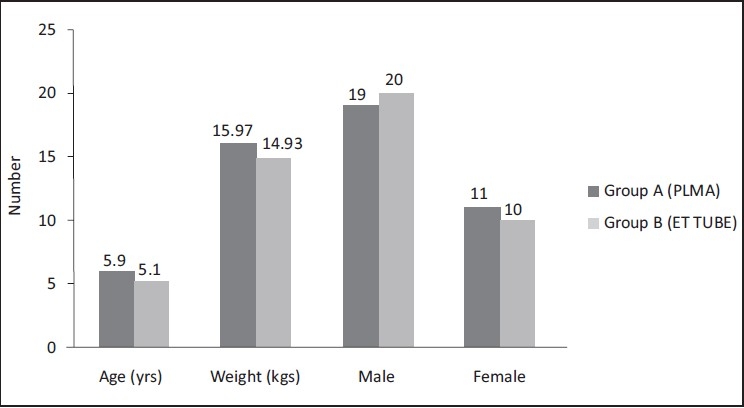
Demographic profile of patients

The patients’ demographic profile were comparable in both the groups [Figure 1]. There was male predominance in both the groups.

The success rate to place the PLMA at first attempt was 83.33% and only 16.67% patients required second attempt. The success rate to intubate the patients was 96.67% and 3.33% at first and second attempt respectively [Table 1].

**Table 1:** Number of attempts at insertion

Author	Attempts	Group PLMA (%)	Group ETT (%)
Self	1^st^	83.33	96.67
Sinha *et al*.	1^st^	88	100
Misra *et al*.	1^st^	88	100
Dave *et al*.	1^st^	93.33	-
Lim *et al*.	1^st^	86	86

PLMA: ProSeal laryngeal mask airway

Haemodynamic responses were lower for the placement of PLMA than the ET intubation. The mean pulse rate (bpm) increased from a baseline value of 103.70±11.56 to 109.50±12.41 and from 102.46±11.46 to 122.83±8.30 after the placement of PLMA and the endotracheal intubation respectively. The increase in the pulse rate was statistically significant (*P*<0.05) in both the groups. The mean pulse rate returned to the base line value after 5 mins of placement of PLMA (Group A). The increase in the pulse rate was statistically significant (*P*<0.05) even after 10 min of endotracheal intubation (Group B) [Table 2]. The increase (%) in pulse rate was higher after ET intubation than after placement of PLMA (*P*<0.05).

**Table 2:** Comparison of mean pulse rate (beats/min) (Mean±S.D.)

Group	Base line	Just after insertion	5 min	10 min
A (PLMA)	102.70±11.56	109.50±12.41	103.56±11.18	99.96±13.15
*P*-value		<0.05	>0.05	>0.05
B (ET tube)	102.46±11.46	122.83±8.30	113.00±14.94	110.26±15.68
*P*-value		<0.05	<0.01	<0.05

PLMA: ProSeal laryngeal mask airway

The increase in mean SBP from the baseline after insertion of PLMA or ET was statistically insignificant (*P*>0.05) in both group A and group B. There was a statistically significant (*P*<0.05) decrease in mean SBP (mmHg) 97.86±8.46 from the baseline value of 105.86±9.78, 5 min after placement of PLMA (Group A). The mean SBP (mmHg) 98.26±11.68 also decreased from the baseline mean SBP of 103.60±12.46, 5 min after ET intubation (Group B) (*P*>0.05).

The increase in mean DBP and MBP was statistically insignificant (*P*>0.05) in both group A and group B. There was a statistically significant (*P*<0.05) decrease in mean DBP and MBP after 5 min of insertion of respective devices in both groups [Table 3].

**Table 3:** Comparison of mean blood pressure (mm Hg) (Mean±S.D.)

Group	Base line	Just after insertion	5 min	10 min
A (PLMA)	79.76±9.95	79.86±9.36	71.66±9.62	69.93±8.25
*P*-value		>0.05	<0.05	<0.01
B (ET tube)	75.51±12.79	77.46±14.39	67.43±11.27	73.10±12.62
*P*-value		>0.05	<0.05	>0.05

PLMA: ProSeal laryngeal mask airway, ET: Endotracheal tube

There was no significant difference in mean SpO_2_ (%) and EtCO_2_ level recorded at different time intervals between the two groups (*P*>0.05).

There was a significant incidence of cough in group B (30%) patients after extubation as compared with the group A (6.6%) patients (PLMA) (*P*<0.05). Bronchospasm was seen in two (6.6%) patients after extubation in group B but none of the patients in the group A after removal of PLMA. After removal of PLMA (Group A), blood on the posterior surface of PLMA was noted in six (20%) cases but only in two (6.6%) cases blood on ET tube was observed after extubation (Group B). There was no incidence of aspiration in either group. There was no incidence of hoarseness or sore throat after removal of PLMA or ETT and postoperatively even after 24 h [Table 4].

**Table 4:** Perioperative complications

Complications	Group A (PLMA)		Group B (ET tube)	
	No.	%	No.	%
Cough	2	6.6	9	30
Laryngospasm	0	0	0	0
Bronchospasm	0	0	2	6.6
Blood on device	6	20	2	6.6
Aspiration	0	0	0	0
Hoarseness/sore throat	0	0	0	0

PLMA: ProSeal laryngeal mask airway, ET: Endotracheal tube

## DISCUSSION

The LMA^™^ and other supraglottic airways have radically changed paediatric anaesthesia practice and have become a key component of airway management in children.[1] There are limitations of Classic LMA (air leak, gastric distension and aspiration). In ProSeal LMA, there is presence of drain tube, integral bite block[1] and different cuff design, increased depth of the bowl to improve the seal with the larynx,[4] from CLMA.

In our study we found that the endotracheal intubation was done in 96.67% patients at first attempt where as PLMA was inserted in 83.33% patients at first attempt. Sinha *et al*. and Misra *et al*. reported that all patients were intubated at first attempt while the PLMA was placed in 88% patients at first attempt in paediatric and adult laparoscopic surgeries, respectively. Dave *et al*. reported the success rate to place the PLMA in first attempt was 93.33%. Lim *et al*. in gynaecological laparoscopy noted that the numbers of attempts for successful insertion were similar for both PLMA and ET tube (86% and 85%, respectively). Misra *et al*. suggested that laryngoscopy and tracheal intubation are the main forte of a successful anaesthesiologist. Hence, the 100% first attempt success in their study where difficult airways were excluded was an occurrence on expected lines. The different morphology of PLMA from the CLMA and after deflation the semirigid distal end of drain tube[1] of PLMA may contribute to difficult insertion.

After placement of PLMA the patient were haemodynamically more stable than after ET intubation. The PLMA position was confirmed by the gel displacement test, bilateral chest movements and square wave capnography.[6] The haemodynamic responses were observed only for short period of time after PLMA insertion than ET intubation.

The findings in our study are comparable with the study of Dave *et al*. and Misra *et al*.

After extubation there was significant incidence of cough as compared to after removal of PLMA. Maltby *et al*. and Sinha *et al*. also reported that the incidence of cough was higher after extubation.

Bronchospasm was noted in two (6.6%) cases in group B patients and no cases in group A patients. The finding in our study also correlated with the study of other authors.[7]

Supraglottic airways could be less irritating[8] to upper or lower airway and associated with less laryngeal stimulation[7] leading to less significant postoperative complications.

Blood on the posterior surface of PLMA was observed in six (20%) patients in group A but in group B patients only in two (6.6%) cases blood on ET tube was observed after extubation. In these patients there occurred some trauma during laryngoscopy and since uncuffed tubes were used in these patients, probably it may be the reason for this unusual complication. Our findings are comparable with Lim *et al*. who have reported 7% incidence of blood staining on PLMA and 6% in ET tube.[9] In one of the studies blood on the tracheal tube is reported more frequently than the PLMA.[10]

There was no incidence of aspiration in either group of patients during induction of anaesthesia, intraoperative period or after removal of the respective airway device. The other authors[2,11,12] also reported the similar findings.

There was no incidence of hoarseness or sore throat after removal of PLMA or ETT and postoperatively even after 24 h.

## CONCLUSION

Based upon the above observations, results and discussion it is concluded that during routine paediatric surgical procedures of short duration, ProSeal LMA is useful alternative to endotracheal intubation. Though the PLMA is slightly difficult to place, effective airway can be achieved easily by an experienced anaesthesiologist and there are lower incidences of complications.
